# The Melanin-Concentrating Hormone (MCH) System Modulates Behaviors Associated with Psychiatric Disorders

**DOI:** 10.1371/journal.pone.0019286

**Published:** 2011-07-19

**Authors:** Shinjae Chung, Michel M. M. Verheij, Peter Hesseling, Ruben W. M. van Vugt, Mahalah Buell, James D. Belluzzi, Mark A. Geyer, Gerard J. M. Martens, Olivier Civelli

**Affiliations:** 1 Department of Pharmacology, University of California Irvine, Irvine, California, United States of America; 2 Department of Developmental and Cell Biology, University of California Irvine, Irvine, California, United States of America; 3 Department of Pharmaceutical Sciences, University of California Irvine, Irvine, California, United States of America; 4 Department of Molecular Animal Physiology, Donders Institute for Brain, Cognition and Behaviour, and Nijmegen Center for Molecular Life Sciences, Radboud University, Nijmegen, The Netherlands; 5 Department of Psychiatry, University of California San Diego, La Jolla, California, United States of America; University of Queensland, Australia

## Abstract

Deficits in sensorimotor gating measured by prepulse inhibition (PPI) of the startle have been known as characteristics of patients with schizophrenia and related neuropsychiatric disorders. PPI disruption is thought to rely on the activity of the mesocorticolimbic dopaminergic system and is inhibited by most antipsychotic drugs. These drugs however act also at the nigrostriatal dopaminergic pathway and exert adverse locomotor responses. Finding a way to inhibit the mesocorticolimbic- without affecting the nigrostriatal-dopaminergic pathway may thus be beneficial to antipsychotic therapies. The melanin-concentrating hormone (MCH) system has been shown to modulate dopamine-related responses. Its receptor (MCH1R) is expressed at high levels in the mesocorticolimbic and not in the nigrostriatal dopaminergic pathways. Interestingly a genomic linkage study revealed significant associations between schizophrenia and markers located in the MCH1R gene locus. We hypothesize that the MCH system can selectively modulate the behavior associated with the mesocorticolimbic dopamine pathway. Using mice, we found that central administration of MCH potentiates apomorphine-induced PPI deficits. Using congenic rat lines that differ in their responses to PPI, we found that the rats that are susceptible to apomorphine (APO-SUS rats) and exhibit PPI deficits display higher MCH mRNA expression in the lateral hypothalamic region and that blocking the MCH system reverses their PPI deficits. On the other hand, in mice and rats, activation or inactivation of the MCH system does not affect stereotyped behaviors, dopamine-related responses that depend on the activity of the nigrostriatal pathway. Furthermore MCH does not affect dizocilpine-induced PPI deficit, a glutamate related response. Thus, our data present the MCH system as a regulator of sensorimotor gating, and provide a new rationale to understand the etiologies of schizophrenia and related psychiatric disorders.

## Introduction

Prepulse inhibition (PPI) is the phenomenon where a startle response produced by an intense stimulus (pulse) is suppressed when a weak prestimulus (prepulse) immediately precedes it. PPI is observed in many species from laboratory animals to human [1]–[3] and has been used as a behavioral paradigm to measure sensorimotor gating. Significant PPI deficits have been observed in patients with schizophrenia and other psychopathological disorders associated with dopamine (DA) dysregulation [1], [3]–[5]. These deficits in PPI are thought to underlie problems with inhibitory mechanisms in sensorimotor gating, for example sensory overload [4], [6], [7].

The mesocorticolimbic dopaminergic system has been shown to be involved in modulating PPI [3]. For instance, DA infusion into the nucleus accumbens disrupts PPI [8], [9]. PPI deficits induced by psychotomimetic administration can be reversed by antipsychotic drugs [2]. Both typical and atypical antipsychotics reverse apomorphine-induced PPI deficits in rats and their ability to reverse these deficits has been shown to correlate with clinical efficacy and their affinity for D2R [10], [11]. PPI in rodents is thus a useful animal model to predict antipsychotics' efficacy. Other neurotransmitter systems acting independently of the DA system are involved in PPI as well. Most notably, psychotomimetics acting through the glutamate system such as phencyclidine (PCP) and dizocilpine (MK-801) can induce PPI deficits in rodents [2], [12], [13].

Sensorimotor gating varies across individuals that are differentially sensitive to dopaminergic drugs. For instance, rats that have been pharmacogenetically selected for a high susceptibility to apomorphine (so-called APO-SUS rats) show less PPI compared to rats selected for a low susceptibility to apomorphine (so-called APO-UNSUS rats) [14]. This is in line with the finding that APO-SUS rats have a hyperactive mesolimbic dopaminergic system when compared to the APO-UNSUS rats [15], [16]. The APO-SUS rats have been described as an animal model displaying certain aspects of schizophrenia (for review [17]).

Stereotypies represent another symptom observed in people with neuropsychiatric disorders [18], [19]. Stereotypies are repetitive and purposeless behaviors such as head banging and body rocking. They are also found in chronic cocaine or amphetamine users [20]–[22] and in Parkinson's patients treated with a high dose of DA agonists [23], [24] which points to the importance of DA in the etiology of stereotypies. Systemic injections of DA agonists for example amphetamine or apomorphine in laboratory animals induce stereotyped behaviors such as constant sniffing, licking and gnawing [25]. Indeed, efficacy of antipsychotics has often been assessed by measuring their efficacy at reducing stereotypies. Yet because the neuronal target important for stereotypic behavior is the caudate putamen [26], [27], these drugs often induce adverse motor control responses. Finding a neuronal system that modulates the mesolimbic dopaminergic system without interfering with the nigrostriatal system should provide an advantageous basis for antipsychosis intervention. The melanin-concentrating hormone (MCH) system represents a possible candidate.

MCH is a 19 amino acid cyclic peptide which is predominantly expressed in the hypothalamus [28], [29]. In rodents, MCH interacts with one G protein-coupled receptor, MCH1R [30]–[34]. That there may be a link between schizophrenia and the activity of the MCH system has been suggested by a genomic linkage study which revealed significant associations between schizophrenia and a number of SNPs and haplotypes located in the MCH receptor gene locus [35]. MCH1R is expressed in a number of brain regions, but most notably at high levels in the mesocorticolimbic dopamine pathway and at very low levels in the caudate putamen [36]. The MCH system has been shown to be linked to the activities of the dopamine system. In the nucleus accumbens shell (NAcSh), MCH1R colocalizes with dopamine D1 and D2 receptors [37], [38]. MCH potentiates D1R plus D2R agonist-induced cellular firing [37] and decreases D1R agonist-induced GluR1 phosphorylation [38], [39]. MCH1RKO mice are hypersensitive to amphetamine [40], [41] and central MCH injections increase cocaine-induced hyperactivity [37]. On the basis of these in vitro and in vivo data, we hypothesize that the MCH system may modulate the PPI of startle response without affecting stereotypy. We therefore analyzed the effects of MCH on PPI in mice injected with apomorphine or dizocilpine and in the APO-SUS/ APO-UNSUS rat model and its effects on stereotypies.

## Materials and Methods

### Ethics Statements

All experimental procedures were reviewed and approved by the institutional animal care and use committee of University of California Irvine, USA and Radboud University Nijmegen, the Netherlands (IACUC #2002-2343, DEC #2009-222).

### Animals

Male C57BL/6 mice (NCI, Bethesda, MD), age 9–11 wks were used. The generation of the APO-SUS and APO-UNSUS rat lines with a high and low susceptibility for apomorphine, respectively, has been described previously [42], [43]. These rats were bred and reared in the Central Animal Facility of the Radboud University Nijmegen, the Netherlands. Male APO-SUS and APO-UNSUS rats (age 9–12 wks) of the 30^th^ generation of the replicate line were used for both behavioural tests and post-mortem processing of the brain. Both mice and rats were group-housed and maintained on a 12-h light/dark cycle (lights on at 07:00) with food and water available *ad libitum*. Rats were individually housed three days before the PPI or stereotypy measurement to ensure that the behavioral outcome of the animal was not affected by the behavior of their cage mate(s) [14], [43]. All experimental procedures were performed in compliance with (inter)national and institutional guidelines for the care and use of laboratory animals.

### Drugs

Apomorphine or dizocilpine (Sigma-Aldrich, St. Louis, MO) was dissolved in saline solution containing 0.1% ascorbic acid or in saline solution respectively. Apomorphine or dizocilpine was administered subcutaneously or intraperitoneally respectively. Doses for the drugs were chosen based on previous reports (mice [44] and rats [42]). MCH (Invitrogen, Carlsbad, CA) or MCH1R antagonist, TPI 1361-17 [45] was dissolved in PBS containing 0.1% BSA (Fisher Scientific, Pittsburgh, PA) and was given i.c.v. (mice [37], and rats [46]). Mice were slightly anesthetized with isoflurane and MCH or TPI 1361-17 was transcranially injected into the lateral ventricle by a freehand i.c.v. injection (for details: [37]). Rats, which were also anesthetized with isoflurane, were equipped with a unilateral guide cannula (for details: [46]). Correct placement of injections was verified after the experiments by histological examination and animals with misplaced i.c.v. injections were excluded. Vehicle, MCH and/or TPI 1361-17 was injected 5 min before apomorphine or dizocilpine injections. Rats were tested for PPI, followed by exposure to the gnawing box one week later. There was no repeat drug testing in the PPI experiments of both mice and rats. In the first group of rats that was used to test the effects of MCH on PPI, TPI 1361-17 was given before exposure to the gnawing box. In the second group of rats that was used to test the effects of TPI 1361-17 on PPI, MCH was given before exposure to the gnawing box.

### Acoustic Startle Procedure

Startle reactivity was measured according to previously described procedures (for mice [44], for rats [43]) using San Diego Instruments (San Diego, CA) startle chambers and SR-LAB software. Each chamber had a clear nonrestrictive Plexiglas cylinder resting on a platform inside of a ventilated and sound attenuated box. A high frequency loudspeaker inside of each chamber produced background noise of 65 dB for the mice studies and 70 dB for the rat studies as well as the various acoustic stimuli (see below). Vibrations of the Plexiglas cylinder caused by the body startle response of the animal were converted into analog signals by a piezoelectric accelerometer attached to the platform. Calibration was performed every time used to ensure the accuracy of the sound levels and startle measurements.

One week before drug testing, mice underwent a brief baseline session to create treatment groups matched for baseline startle reactivity and PPI response. During this baseline session, the 65 dB background noise was presented for 1 min and continued throughout the remainder of the session. A total of 24 trials were presented (18 of 120 dB pulse-alone trials and 6 of 77 dB prepulse+120 dB pulse trials) in a pseudorandom order.

During testing sessions, mice or rats were placed in the startle chambers and the background noise (see above) was presented for a 5 min acclimatization period and continued throughout the test session. PPI session consisted of startle trials, prepulse trials and no-stimulus trials. Prepulse trials consisted of a 20 msec prepulse, 80 msec delay, followed by a 40 msec (in mice) or 20 msec (in rats) 120 dB startle trial. Prepulse intensities were 3, 6 and 12 dB above the 65 dB background noise in the mice studies and 3, 5 and 10 dB above the 70 dB background noise in the rat studies [43]. The no stimulus trials consisted of background noise only. This represents a control trial for detecting differences in overall activity. Startle trials, prepulse trials and no stimulus trials were presented in a pseudorandom order and there was an average of 15 sec between the trials. Mice and rats were placed into the startle chambers 5 min after drug injection.

The amount of PPI was calculated as a percentage score for each acoustic prepulse intensity: % PPI = 100−{[(startle response for prepulse+pulse trials)/(startle response for pulse-alone trials)]×100}. The magnitude of the response was calculated as the average responses to all of the pulse-alone or prepulse trials.

### In situ hybridization

In situ hybridization was performed similarly as previously described [36], [37] with slight modification. pMCH probe is a kind gift from Dr. Jean-Louis Nahon (Institute de Pharmacologie Moleculaire et Cellulaire, Valbonne, France) and previously described [47]. The probe was digested with either *SmaI* or *HindIII*, and then antisense and sense [^35^S]-uridine 5′-triphosphate (UTP)-labeled riboprobes were synthesized by T7 and T3 RNA polymerases, respectively (Amersham, Arlington Heights, IL). Tissue sections were processed for *in situ* hybridization as previously described with slight modifications. Briefly, 20 µm sections were pretreated with proteinase K (0.1 µg/ml), acetylated, dehydrated through ethanol (50, 70, 95, and 100%), and air dried. Pretreated sections were then incubated for 20 hours at 60°C, with hybridization buffer (50% formamide, 10% dextran sulfate, 0.02% Ficoll, 0.02% polyvinylpyrolidone, 0.02% bovine serum albumin, 500 µg/ml tRNA, 10 mM dithiothreitol, 0.3 M NaCl, 10 mM Tris, pH 8.0, 1 mM EDTA, pH 8.0) containing [^35^S]-UTP labeled sense or antisense riboprobes (5×10^6^ cpm/ml). After the sections were hybridized, they were treated with RNase A (20 µg/ml) for 30 minutes at 37°C and then washed four times in decreasing salinity (from 2× to 0.1× standard saline citrate [SSC] buffer) and a 30-minute wash at 68°C. Next, sections were dehydrated through ethanol (50, 70, 95, and 100%), air dried, and exposed to MR-1 Kodak film for 2 hrs. Sections were processed further for cresyl violet staining. Autoradiographic images were quantified using a computer-based image analysis system (MCID, Image Research Inc., St Catharines, ON, Canada). Brain areas on autoradiograms were identified with reference to adjacent brain sections processed for cresyl violet staining. MCH cells lateral to the fornix were considered to be in the LH. Corresponding sections were compared between APO-UNSUS and APO-SUS rats. Optical densities in brain regions were measured (Figure S4) and the corresponding values of radioactivity were determined by interpolation from a standard curve, generated from C14 standards (American Radiolabeled Chemicals, St Louis, MO). The values obtained represent the average of measurements taken from 6 sections per animal (one final data point per animal) in each brain site.

### Stereotypy measurement in mice

Mice were individually placed in a new cage without bedding and were allowed to habituate to this environment for 30 min. These animals were then injected either with vehicle or MCH (i.c.v.) 10 min prior to the apomorphine (s.c.) injection. At 5 min after the apomorphine injection, stereotyped behaviors were observed and recorded by an observer blind to the treatments for 10 sec every minute for 30 min. Stereotypy rating scale was slightly modified from a rating scale used by LaHoste and Marshall [48]. 0 = inactivity, 1 = grooming, 2 = locomotion, 3 = sniffing directed upward, 4 = sniffing with head down, 5 = intense sniffing in a small circumscribed area, 6 = intense sniffing with bursts of lick, 7 = constant licking or gnawing box, 8 = self lick or biting. Rating scores for 30 min were collapsed and shown as a total stereotypy counts.

### Stereotypy measurement in rats

APO-SUS and APO-UNSUS rats were allowed to habituate to the test room for 30 min and were then injected either with vehicle or 10 nmole of MCH (i.c.v.) or 10 nmole of TPI 1361-17 (i.c.v.) 10 min prior to a systemic injection of 1.5 mg/kg of apomorphine (s.c.). At 5 min after the apomorphine injection, stereotyped behaviors were scored in a so-called gnawing box (for details see [42]). This box was slightly modified from the box described by Ljungberg and Ungerstedt [49] and contains 32 holes surrounded by concentric ridges to promote stereotypic gnawing behavior. All rats were placed in this box for 45 min and the total gnawing count was automatically recorded.

### Data analysis

**Figure 1:**
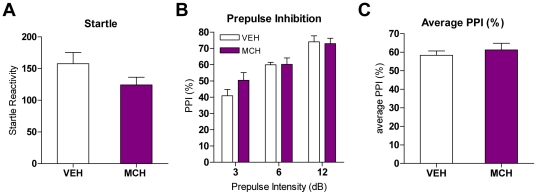
MCH effects on PPI in mice. A. Startle reactivities after vehicle or MCH (1 nmole) injection in mice (n = 8–9). Values represent mean startle reactivity ± SEM. B. PPI levels in mice after MCH (1 nmole) injection (n = 8–9). Values represent mean % PPI ± SEM. C. Average of PPI values after MCH injections in mice. Values represent average of % PPI elicited by three prepulse intensities ± SEM.

**Figure 2:**
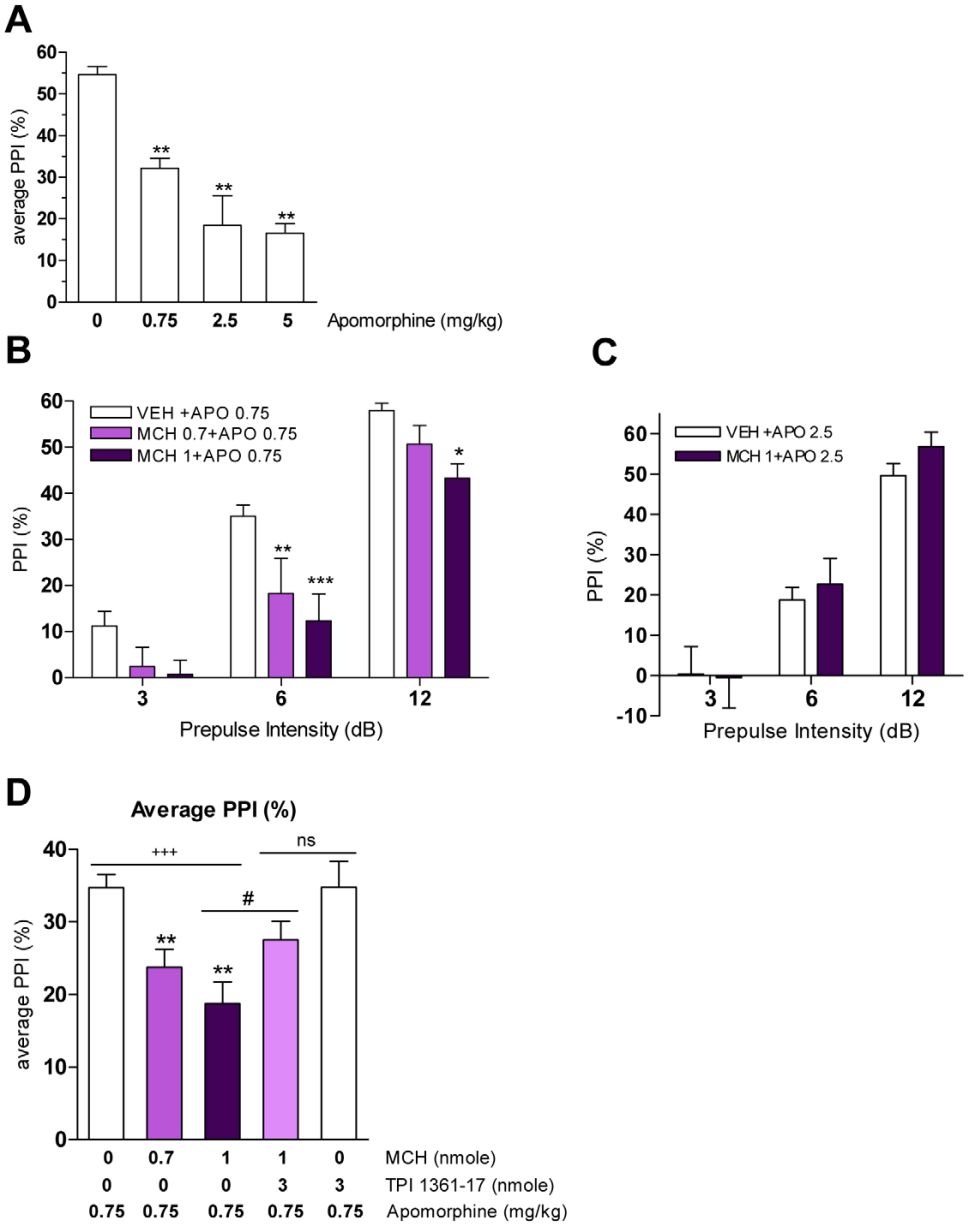
MCH effects on apomorphine-induced PPI deficits in mice. A. Effect of apomorphine (0, 0.75, 2.5, 5 mg/kg) on PPI (**p<0.01 vs. VEH, one-way ANOVA, with Dunnett's test; n = 5–21). Values represent average of % PPI elicited by three prepulse intensities ± SEM. B. PPI after MCH pretreatment (0, 0.7, 1 nmole) in apomorphine (0.75 mg/kg)-treated mice (*p<0.05, **p<0.01, ***p<0.001 vs. VEH+APO 0.75, two-way ANOVA with Bonferroni test; n = 8–19). C. PPI after MCH pretreatment (0, 1 nmole) in apomorphine (2.5 mg/kg)-treated mice (n = 8). Values (B–C) represent mean % PPI ± SEM. D. Average of PPI values after MCH and/or TPI 1361-17 pretreatment in apomorphine-treated mice (^+++^ *p*<0.001, dose effect, one-way ANOVA; **p<0.01 vs. APO 0.75, one-way ANOVA with Dunnett's test; ^#^ *p*<0.05 vs. APO 0.75+MCH1, t-test; ^ns^ *p*>0.05 vs. APO 0.75+MCH1+TPI 3, t-test; n = 8–19). Values represent average of % PPI elicited by three prepulse intensities ± SEM.

**Figure 3:**
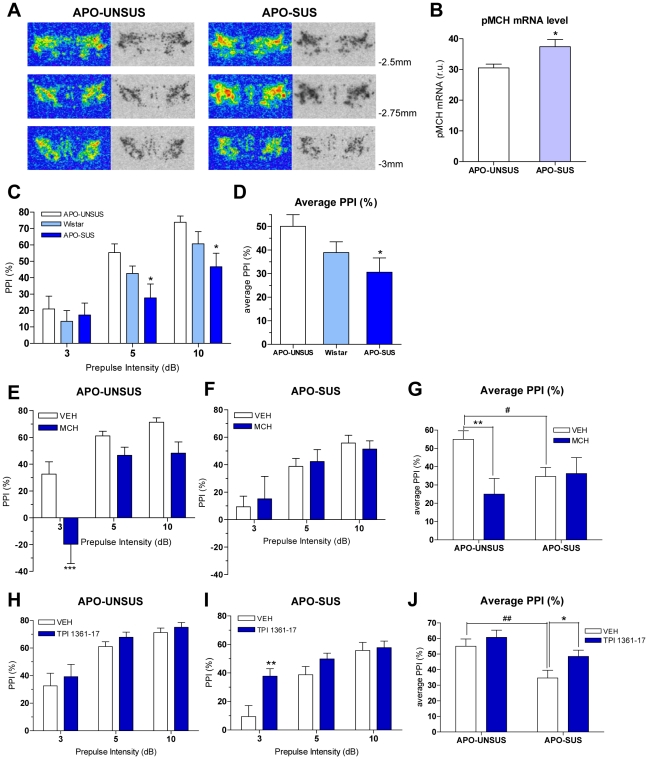
MCH effects on PPI in APO-UNSUS and APO-SUS rats. A. Autoradiographic images illustrating the pMCH expression patterns in hypothalamic areas of APO-UNSUS and APO-SUS rats approximately at −2.5, −2.75 and −3 mm from bregma. B. pMCH mRNA levels in the lateral hypothalamus (LH) at −2.75 from bregma of APO-UNSUS and APO-SUS rats (*p<0.05 vs. APO-UNSUS, t-test; n = 5). C. PPI levels of naïve APO-UNSUS, wild type Wistar and APO-SUS rats (*p<0.05 vs. APO-UNSUS, two-way ANOVA with bonferroni test; n = 15). Values represent mean % PPI ± SEM. D. Average PPI level of naïve APO-UNSUS, wild type Wistar and APO-SUS rats (*p<0.05 vs. APO-UNSUS, one-way ANOVA with bonferroni test; n = 15). Values represent average of % PPI upon three prepulse intensities ± SEM. E. Effect of MCH (10 nmole) on PPI in APO-UNSUS rats (***p<0.001 vs. VEH, two-way ANOVA with Bonferroni test; n = 10–13). F. Effect of MCH (10 nmole) on PPI in APO-SUS rats (n = 10–13). G. Average of PPI values after VEH or MCH (10 nmole) injections in APO-UNSUS and APO-SUS rats (**p<0.01, ^#^ *p*<0.05 vs. VEH-treated APO-UNSUS rats, two-way ANOVA with Bonferroni test; n = 10–13). H. Effect of TPI 1361-17 (10 nmole) on PPI in APO-UNSUS rats (n = 12–13). I. Effect of TPI 1361-17 (10 nmole) on PPI in APO-SUS rats (**p<0.01 vs. VEH, two-way ANOVA with Bonferroni test; n = 13). J. Average of PPI values after VEH or TPI 1361-17 (10 nmole) injections in APO-UNSUS and APO-SUS rats (^##^ *p*<0.01 vs. VEH-treated APO-UNSUS, *p<0.05 vs. VEH-treated APO-SUS, two-way ANOVA with Bonferroni test; n = 12–13). Data (E,F,H,I) are expressed as mean % PPI ± SEM. Data (G,J) represent average of % PPI elicited by three prepulse intensities ± SEM.

**Figure 4:**
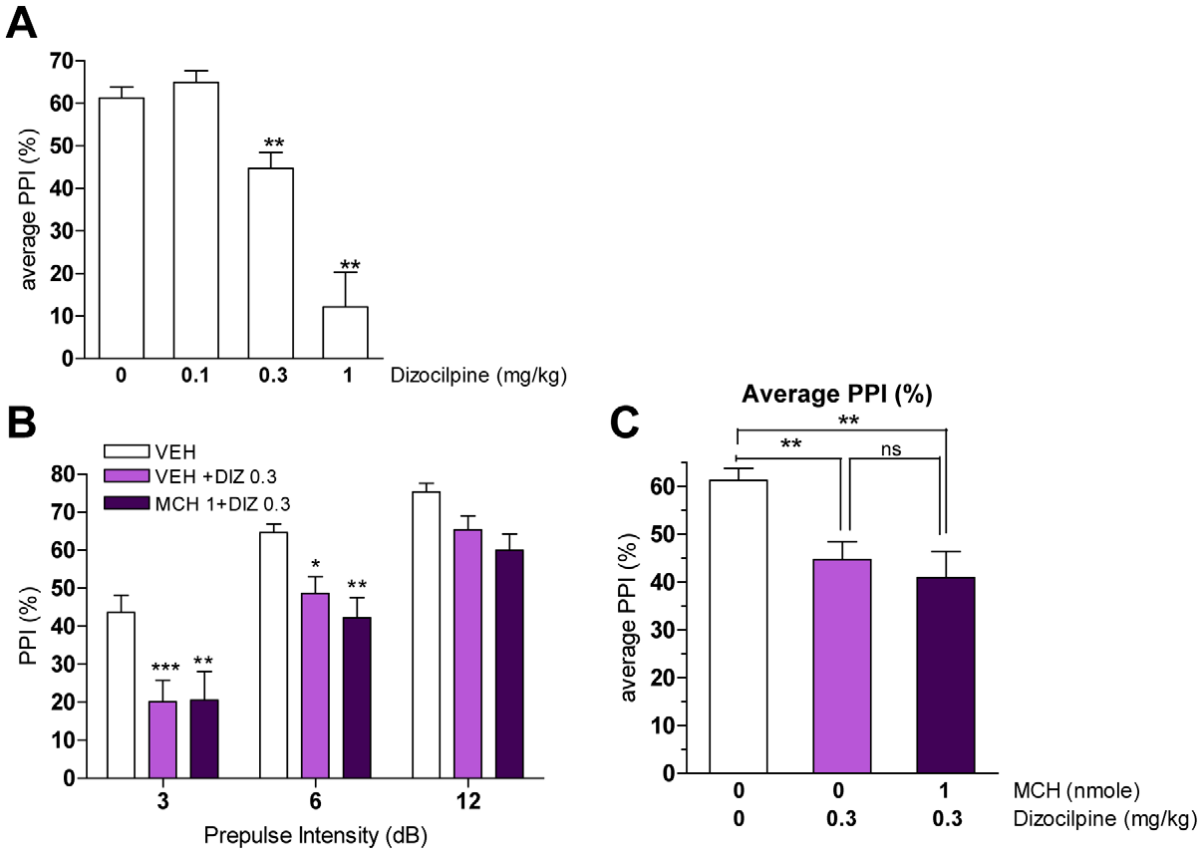
MCH effects on dizocilpine-induced PPI deficits in mice. A. Effect of dizocilpine (0, 0.1, 0.3, 1 mg/kg) on PPI (**p<0.01 vs. VEH, one-way ANOVA, Dunnett's test; n = 5–13). Values represent average of % PPI upon three prepulse intensities ± SEM. B. PPI levels after MCH pretreatment (0, 1 nmole) in dizocilpine (0, 0.3 mg/kg)-treated mice (*p<0.05, **p<0.01, ***p<0.001 vs. VEH, two-way ANOVA with Bonferroni test; n = 8–12). Values represent mean % PPI ± SEM. C. Average of PPI value after MCH pretreatment on dizocilpine injections (**p<0.01 vs. VEH, ^ns^ *p*>0.05 vs VEH+DIZ0.3, one-way ANOVA with Bonferroni test; n = 8–12). Values represent average of % PPI from three prepulses ± SEM.

**Figure 5:**
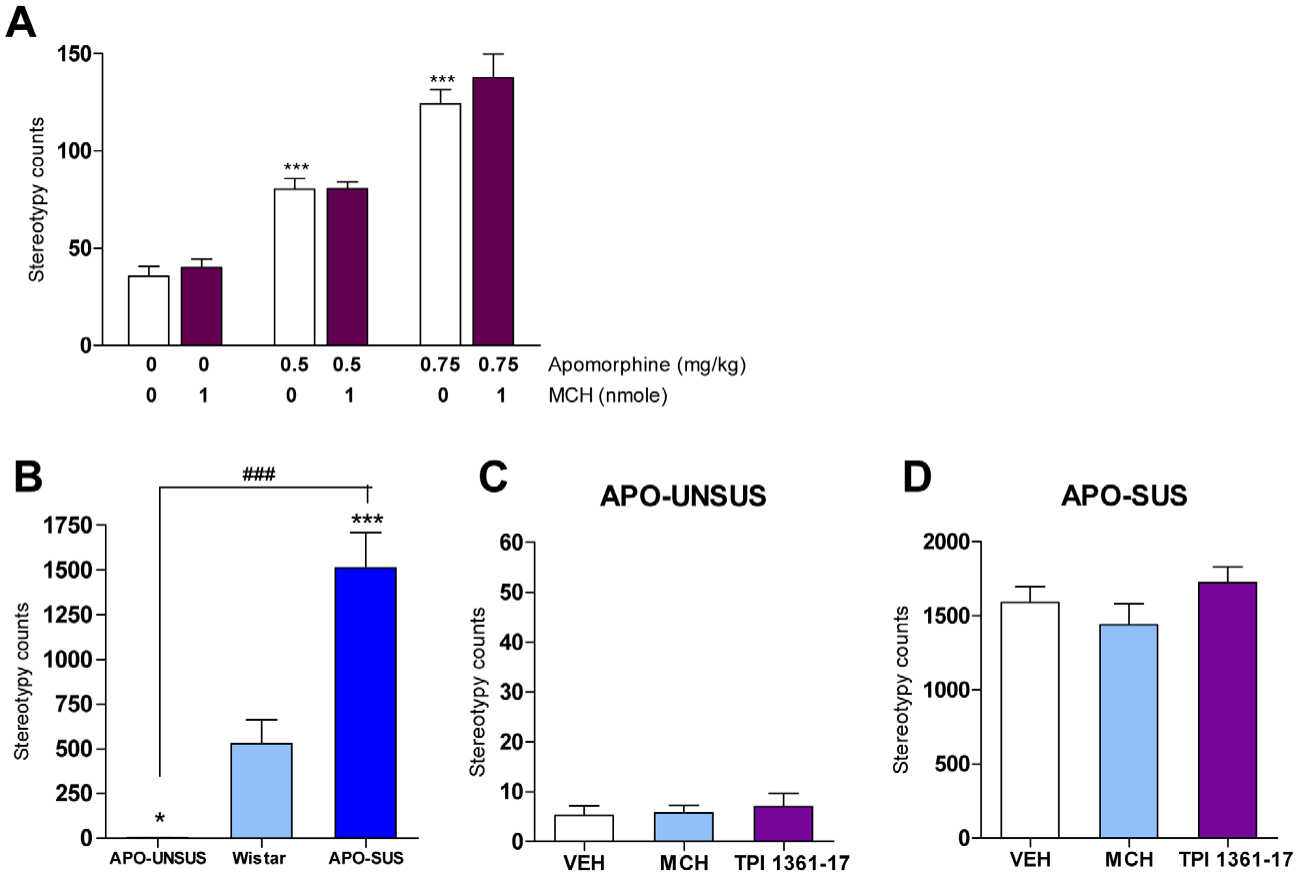
Apomorphine-induced stereotyped behaviors upon MCH injection. A. Effect of MCH (0, 1 nmole) on apomorphine (0, 0.5, 0.75 mg/kg)-induced stereotyped behaviors in mice (***p<0.001 vs. VEH, two-way ANOVA with Bonferroni test; n = 5–10). B. Stereotypy counts in APO-UNSUS, wild type Wistar and APO-SUS rats after apomorphine (1.5 mg/kg) injections (*p<0.05 ***p<0.001 vs Wistar ^###^ *p*<0.001 vs APO-UNSUS, one-way ANOVA with bonferroni's posttests; n = 10–15). C. Effect of MCH (10 nmole) and TPI 1361-17 (10 nmole) on apomorphine (1.5 mg/kg)-induced stereotyped behavior in APO-UNSUS rats (n = 10–13). D. Effect of MCH (10 nmole) and TPI 1361-17 (10 nmole) on apomorphine (1.5 mg/kg)-induced stereotyped behavior in APO-SUS rats (n = 11–13). Values (A–D) represent total stereotypy counts ± SEM.

Prism software (GraphPad, San Diego, CA) was used for statistical analysis. Data was expressed as mean ± SEM. Results were analyzed by t-test or ANOVA followed by the appropriate post hoc comparisons and *p*<0.05 was considered statistically significant. In PPI analysis of mice (Figure 1, 2, 4), repeated-measures two-way ANOVA with bonferroni post hoc tests was used with treatment, a between-subjects variable and prepulse intensity, a within-subjects variable. PPI values were also shown as average PPI (%) of the three prepulse intensities and analyzed using one-way ANOVA with Dunnett's test or t-test. In PPI analysis of APO-SUS/UNSUS rats (Figure 3), repeated-measures two-way ANOVA with bonferroni post hoc tests was used with treatment, a between-subjects variable and prepulse intensity, a within-subjects variable. PPI values were also shown as average PPI (%) of the three prepulse intensities and compared between genotypes. Here, two-way ANOVA with bonferroni post hoc tests was used and genotype and treatment were between-subjects variables. In stereotypy analysis of mice (Figure 5), two-way ANOVA with bonferroni post hoc tests was used with apomorphine dose and MCH dose, between-subjects variables. F values shown in the result section indicate main effect of treatment or genotype as described on the text unless indicated as genotype×treatment interaction analysis. For brevity, the main effects of prepulse intensity are not being discussed since they were always significant.

## Results

### Startle and PPI responses in mice subjected to MCH

MCH was tested for its effects on startle responses and PPI. Mice were injected with vehicle or MCH (1 nmole) and then subjected to sessions comprised of pulse-alone trials (p120) and prepulse+pulse trials (pp3p120, pp6p120, pp12p120). MCH had no effect on startle (Figure 1A). The inhibition of the startle response by the prepulse was measured and expressed as percent of PPI. PPI increased with increasing prepulse intensities (Figure 1B) and MCH had no effect on PPI levels (Figure 1B,C) when compared to the vehicle-injected group.

### Role of the MCH system in modulating apomorphine-induced PPI deficit in mice

Because we have shown that activation of the MCH system potentiates DA-induced cell firing and cocaine-induced hyperactivity [37], we investigated whether MCH affects apomorphine-induced PPI deficits. The mixed D1/D2 agonist, apomorphine disrupted PPI dose-dependently (Figure 2A) [50]. At first, an intermediate dose of apomorphine, 0.75 mg/kg, was chosen to test the effect of variable amounts of MCH. Vehicle or MCH (0.7 and 1 nmole) was injected prior to apomorphine injections. We found that MCH dose-dependently increases apomorphine-induced PPI deficits (F_[2,32]_ = 13.54, *p*<0.0001; Figure 2B) as also shown as an average of the PPI values upon 3 prepulse intensities (F_[2,32]_ = 13.54, *p*<0.0001; Figure 2D). The effect of MCH was inhibited by co-administration of a specific MCH1R antagonist, TPI 1361-17 (3 nmole) (*p* = 0.0378; Figure 2D), confirming that the MCH effects are MCH1R specific. Pretreatment by TPI 1361-17 alone did not affect the apomorphine-induced PPI disruption (Figure 2D). Apomorphine at higher doses (2.5 mg/kg) exerted an enhanced PPI deficit when compared to the 0.75 mg/kg dose which reached saturating effects (Figure 2A). At this dose, MCH could not further increase the PPI deficit (Figure 2C). This is further evidenced when the effects of MCH (1 nmole) were compounded as a function of apomorphine concentration. MCH decreased PPI level at lower apomorphine concentrations (F_[2,23]_ = 6.65, *p* = 0.0053; Figure S1) but this response reaches a ceiling effect at high doses (Figure 2C).

Startle reactivity levels after drug injections were also examined. Apomorphine significantly decreased startle reactivity at 2.5 mg/kg, but not at 0.75 mg/kg (Table S1). Additional injections of MCH and/or the MCH1R antagonist, TPI 1361-17 prior to apomorphine did not affect startle reactivity.

### Role of the MCH system in modulating PPI in APO-SUS and APO-UNSUS rats

Since MCH increased apomorphine-induced PPI deficits in mice, we examined its role in two outbred rat lines that have different susceptibility to apomorphine, the APO-SUS and APO-UNSUS strains of Wistar rats. We first analyzed mRNA expression levels of the MCH precursor (pMCH) by in situ hybridization in the lateral hypothalamus (LH), the central site of MCH synthesis (Figure 3A,B). We found that the level of pMCH mRNA expression was significantly higher in the LH region (near −2.75 mm from bregma) of APO-SUS rats than that of APO-UNSUS rats (Figure 3A,B). Then, APO-SUS and APO-UNSUS rats were compared to inbred wild type Wistar rats for their responses to PPI (Figure 3C,D). Wistar rats displayed an intermediate PPI phenotype when compared to APO-UNSUS or APO-SUS rats but this was non-significant. APO-SUS rats however exhibited a significant disrupted PPI when compared to APO-UNSUS rats (*p<0.05 vs. APO-UNSUS; Figure 3C,D) (see also [14]). We therefore proceeded by comparing APO-SUS and APO-UNSUS rats to each others.

In view of the higher pMCH levels found in APO-SUS *versus* APO-UNSUS rats, we tested whether administration of the MCH1R antagonist TPI 1361-17 affects PPI in these rats (Figure 3H–J). TPI 1361-17 (10 nmole) did not change PPI in APO-UNSUS rats (Figure 3H) but significantly increased PPI in APO-SUS rats (F_[1,24]_ = 4.72, *p* = 0.04; prepulse intensity×treatment, F_[2,48]_ = 5.28, *p* = 0.008; Figure 3I). Noteworthy, TPI 1361-17 did not affect startle responses in APO-UNSUS and APO-SUS rats (Figure S2B). Conversely, we tested whether MCH injection can decrease PPI in APO-UNSUS rats. Central injections of MCH differentially affected PPI in these two lines of rats (genotype×MCH treatment F_[1,42]_ = 5.73, *p* = 0.02; genotype×prepulse intensity×MCH treatment F_[2,84]_ = 3.41, *p* = 0.04; Figure 3G). MCH (10 nmole) significantly reduced PPI in APO-UNSUS rats (F_[1,21]_ = 10.56, *p* = 0.004; prepulse intensity×treatment, F_[2,42]_ = 6.30, *p* = 0.004; Figure 3E), but not in APO-SUS rats (Figure 3F). APO-SUS rats were also found to exhibit higher startle reactivity relative to APO-UNSUS rats (F_[1,42]_ = 9.36, *p* = 0.004; Figure S2A) (See also [14]). Importantly, MCH injection did not affect startle reactivity in APO-UNSUS and APO-SUS rats (Figure S2A).

### Role of the MCH system in modulating dizocilpine-induced PPI deficit in mice

Dizocilpine (MK-801) is a non-competitive NMDA receptor antagonist that has been shown to also disrupt PPI [50] yet through a mechanism distinct from that of the DA agonist, apomorphine [44]. We analyzed whether acute MCH injection could modulate dizocilpine-induced PPI deficit using the same paradigm that was used for apomorphine. Dizocilpine dose-dependently disrupted PPI (Figure 4A). The intermediate dose of dizocilpine, 0.3 mg/kg, was chosen to test the effect of MCH. Mice were injected either with vehicle or MCH (1 nmole) prior to dizocilpine injection. Dizocilpine significantly disrupted PPI (F_[2,29]_ = 7.64, *p* = 0.002; Figure 4B) but MCH did not significantly affect the dizocilpine-induced PPI deficit as further shown when the percent of PPI is compounded as an average of the PPI values upon 3 prepulse intensities (Figure 4C).

### Role of the MCH system in modulating apomorphine-induced stereotyped behaviors in mice and rats

Apomorphine induces stereotyped behaviors in laboratory animals. To study whether acute activation of the MCH system affects apomorphine-induced stereotypies, vehicle or MCH (1 nmole) and apomorphine (0, 0.5, 0.75 mg/kg) were injected into mice. The animals were observed and recorded every min for a total of 30 min. Stereotyped behaviors were scored using a rating scale [48]. MCH alone did not induce any stereotyped behaviors (Figure 5A). As expected, apomorphine dose-dependently induced stereotyped behaviors (F_[2,39]_ = 83.12, *p*<0.0001; Figure 5A). However, MCH had no effect on this induction (Figure 5A).

To study whether the MCH system modulates apomorphine-induced stereotyped behavior in rats that are selectively bred for a differential stereotyped response to apomorphine, the effect of MCH was also tested in APO-SUS and APO-UNSUS rats. As expected, apomorphine strongly increased stereotyped gnawing in APO-SUS rats but not in APO-UNSUS rats (genotype×apomorphine treatment F_[1,36]_ = 56.72, *p*<0.0001; Figure S3A,B) (See also [17]). The gnawing scores of APO-SUS and APO-UNSUS rats were significantly different from those of wild type Wistar rats (Figure 5B). Intriguingly, neither MCH nor TPI 1361-17 changed stereotyped behavior in both types of rats (Figure 5C,D).

## Discussion

The MCH system has been shown to regulate DA-related responses [37], [38], [40]. The MCH receptor is highly expressed in the limbic part of the brain where DA receptors are predominantly expressed such as the nucleus accumbens shell (NAcSh) and the prefrontal cortex (PFC) [34], [36], [47]. The MCH receptor is expressed at low levels in the caudate putamen [36], which suggests that it may be able to modulate the dopamine tone selectively in the mesocorticolimbic system. The MCH receptor is co-localized with the dopamine D1 and D2 receptors in the NAcSh [37], [38]. MCH receptor antagonists not only reduce food intake, but are also anxiolytic, antidepressant and inhibit cocaine reward [37], [51]–[53]. Furthermore, a human linkage analysis has indicated a possible association between the MCH1 receptor locus and schizophrenia and bipolar disorders [35]. Therefore, we hypothesized that the MCH system might be involved in another dopamine-related response, sensorimotor gating and investigated this hypothesis by using the PPI behavioral paradigm in two rodent systems, apomorphine-treated mice and apomorphine-susceptible rats that were previously found to have disrupted PPI [14].

We show that central injections of MCH in mice do not affect startle or PPI responses. However, because we have previously shown that MCH alone does not induce cellular firing in the NAcSh neurons, but potentiates dopamine D1 plus D2 agonist-induced cellular firing [37], we hypothesized that MCH may affect PPI in mice when it is combined with a dopamine agonist. We therefore used the D1/D2 agonist apomorphine, which is known to induce PPI deficits. First, we found that MCH enhances apomorphine-induced PPI deficits dose dependently. This enhancement is seen at low doses of apomorphine (0.75 mg/kg) and is not detected at saturating doses (2.5 mg/kg) which indicates that MCH can potentiate the effects of low concentrations of apomorphine but cannot increase the effects of saturating doses of apomorphine. This effect was abolished by co-injection of the specific MCH1R antagonist, TPI 1361-17. Thus, MCH is able to potentiate apomorphine-induced PPI deficits, in a manner similar to its effects on DA-induced NAcSh firing and on cocaine-induced hyperactivity [37]. It is important to note that MCH was found to enhance apomorphine-induced PPI deficits without affecting startle reactivity.

We then extended these studies by using the APO-SUS and APO-UNSUS rat model. These animals have been selected and bred to exhibit differences in their susceptibility to apomorphine [42]. APO-SUS rats display a more responsive accumbal catecholaminergic system [15], [16] and a decreased PPI [14] when compared to APO-UNSUS rats. This difference is consistent with the higher levels of dopamine found in the NAc of novelty exposed APO-SUS rats when compared to that of APO-UNSUS rats [15]. These outbred rats represent a model in which differences in the dopamine tone exist naturally and they are therefore useful to evaluate the effects of modulators of the dopamine system (for review [42]). First, we found significantly higher pMCH mRNA levels in the LH of APO-SUS versus APO-UNSUS rats, a first indication that the MCH system may be involved in the phenotypic differences exhibited by these rat strains. The fact that LH neurons project to the mesocorticolimbic pathway [36], [47] is a further indication that the MCH system may modulate PPI differently in the two strains. Indeed, when MCH was administered to both rat strains, it disrupted PPI only in APO-UNSUS rats that exhibit lower pMCH levels. This effect is in line with the MCH potentiating effects we found in mice. On the other hand, the fact that MCH injection did not disrupt PPI in the APO-SUS rats is in line with their high basal pMCH levels and the hyperdopaminergic activity of their mesolimbic DA pathway [15], [16] which may not be increased further by exogenous MCH injection. The apparent discrepancy between the MCH effects in apomorphine-untreated mice and APO-UNSUS rats may be related to species differences such as these found in dopamine levels (as inferred by tyrosine hydroxylase levels [54], [55]) and MCH levels [56] and will need to be analyzed further. Most importantly, when the MCH system was blocked by the antagonist TPI 1361-17, significant increases in PPI were found in APO-SUS but not in APO-UNSUS rats. This correlates well with the higher pMCH mRNA levels found in the LH of APO-SUS rats and with our hypothesis that only activated dopamine systems can be modulated by MCH.

Noncompetitive NMDA receptor antagonists such as PCP and dizocilpine (MK-801) disrupt PPI [2] independently of the DA system because neither D1 nor D2 antagonists reverse PCP or dizocilpine-induced PPI deficits [12], [13], and since dizocilpine disrupts PPI in both D1R KO mice and D2R KO mice [44]. We investigated whether the MCH system modulates the PPI disrupted by dizocilpine. MCH injections did not change the dizocilpine-induced PPI deficit. Our data therefore indicate that the MCH system regulates the PPI modulated by the activity of the DA system, but not by the activity of the NMDA receptor-involved glutamate system.

Stereotyped behaviors are also regulated by the DA system. Indeed, the efficacy of D2R–related antipsychotics has been commonly tested against apomorphine-induced stereotypies. These responses are however thought to rely on DA receptors in the caudate putamen, a region important in the regulation of motor activity [26], [27]. In mice, MCH alone did not induce any stereotypies and had no significant effect on apomorphine-induced stereotypies. This result is consistent with the low level of MCH1R expression found in the caudate putamen [36], a level that is apparently not sufficient to potentiate an apomorphine-induced response as it does in the mouse nucleus accumbens, the region that regulates PPI [8], [9]. This finding is also consistent with the finding that MCH1R KO mice exhibit behavioral differences that are mostly associated with the mesolimbic dopamine system [40], [41], [57]. In line with our findings in mice, the MCH system did not modulate stereotyped behaviors in both APO-UNSUS and APO-SUS rats.

In summary, we show that in mice the MCH system acutely increases apomorphine-induced PPI disruption, but does not affect dizocilpine-induced PPI deficits. In rats, a strain which exhibit higher PPI deficits (APO-SUS) displays significantly higher pMCH mRNA levels. MCH administration decreases PPI in the strain with lower PPI deficit (APO-UNSUS) but not in APO-SUS rats. Moreover blockade of the MCH system increases PPI in APO-SUS rats, but not in APO-UNSUS rats. On the other hand, the MCH system does not affect apomorphine-induced stereotypies in mice and rats. Taken together these data lead us to conclude that the MCH system potentiates dopamine-related responses selectively and spatially, but does not modulate the related glutamate-directed PPI response nor the nigrostriatal system-associated stereotypies. Thus, the MCH system may be targeted for the development of drugs for neuropsychiatric disorders that are related to the overactivity of the mesolimbic DA system.

## Supporting Information

Figure S1 **Percent change of MCH-induced PPI upon increasing doses of apomorphine.** Percent changes of PPI level as function of apomorphine concentrations (0, 0.4 and 0.75 mg/kg) in the presence of MCH (1 nmole). Values represent percent changes of PPI at each dose of apomorphine (^##^ *p*<0.01, dose effect, one-way ANOVA; **p<0.01 vs. APO 0+MCH1, one-way ANOVA with Dunnett's test; n = 8–9).
(TIF)

Figure S2 **Startle reactivities of APO-UNSUS and APO-SUS rats upon MCH or MCH1R antagonist (TPI 1361-17) injections.** A. Startle reactivity of APO-UNSUS and APO-SUS rats upon vehicle or MCH injections (two-way ANOVA, genotype effect F_[1,42]_ = 9.362 *p* = 0.0039, treatment effect F_[1,42]_ = 1.625 *p* = 0.2095; bonferroni test *p<0.05 vs. VEH treated APO-UNSUS; n = 10–13). B. Startle reactivity of APO-UNSUS and APO-SUS rats upon vehicle or TPI 1361-17 injections (two-way ANOVA, genotype effect F_[1,47]_ = 10.77 *p* = 0.0019, treatment effect F_[1,47]_ = 3.962 *p* = 0.0524; n = 12–13). Values represent mean startle reactivity ± SEM.
(TIF)

Figure S3 **Stereotyped behaviors in APO-UNSUS and APO-SUS rats.** Individual differences in apomorphine-induced stereotyped gnawing behaviors in APO-UNSUS and APO-SUS rats (two-way ANOVA, genotype×apomorphine effect: F_[1.36]_ = 56.72, *p*<0.0001; rat type separated into Figure S3A and S3B due to large differences in their Y-axis). A. Stereotypy counts in APO-UNSUS rats upon veh or apomorphine (1.5 mg/kg) injections (Bonferroni posttests, apomorphine effect *p*>0.05). B. Stereotypy counts in APO-SUS rats upon veh or apomorphine (1.5 mg/kg) injections (Bonferroni posttests, apomorphine effect ***p<0.001). Values (A–B) represent total stereotypy counts ± SEM.
(TIF)

Figure S4 **Stereotaxic coordinates of lateral hypothalamus for pMCH mRNA quantification.** Red circles indicate lateral hypothalamic region which was used to quantify pMCH mRNA levels.
(TIF)

Table S1 **Startle reactivities upon apomorphine and/or MCH/MCH1R antagonist (TPI 1361-17) injections.**
(TIF)
