# Supplementary figures and images for: The Melanin-Concentrating Hormone (MCH) System Modulates Behaviors Associated with Psychiatric Disorders
Source: PLoS One. 2011 Jul 19;6(7):e19286. doi: 10.1371/journal.pone.0019286 (PMC3139593; doi:10.1371/journal.pone.0019286)

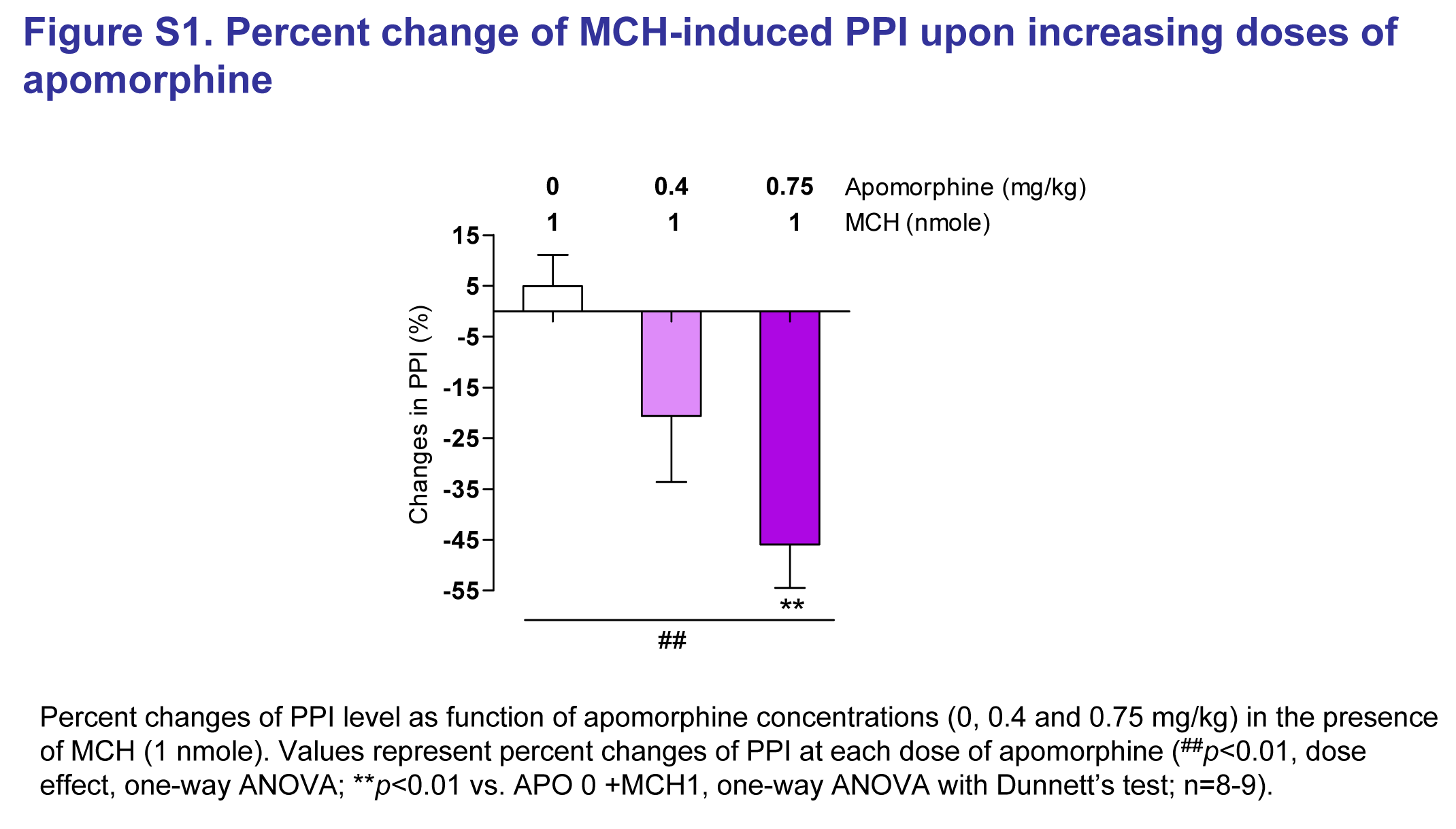

Supplement: Figure S1 — Percent change of MCH-induced PPI upon increasing doses of apomorphine. Percent changes of PPI level as function of apomorphine concentrations (0, 0.4 and 0.75 mg/kg) in the presence of MCH (1 nmole). Values represent percent changes of PPI at each dose of apomorphine (## p<0.01, dose effect, one-way ANOVA; **p<0.01 vs. APO 0+MCH1, one-way ANOVA with Dunnett's test; n = 8–9). (TIF) [file pone.0019286.s001.tif]

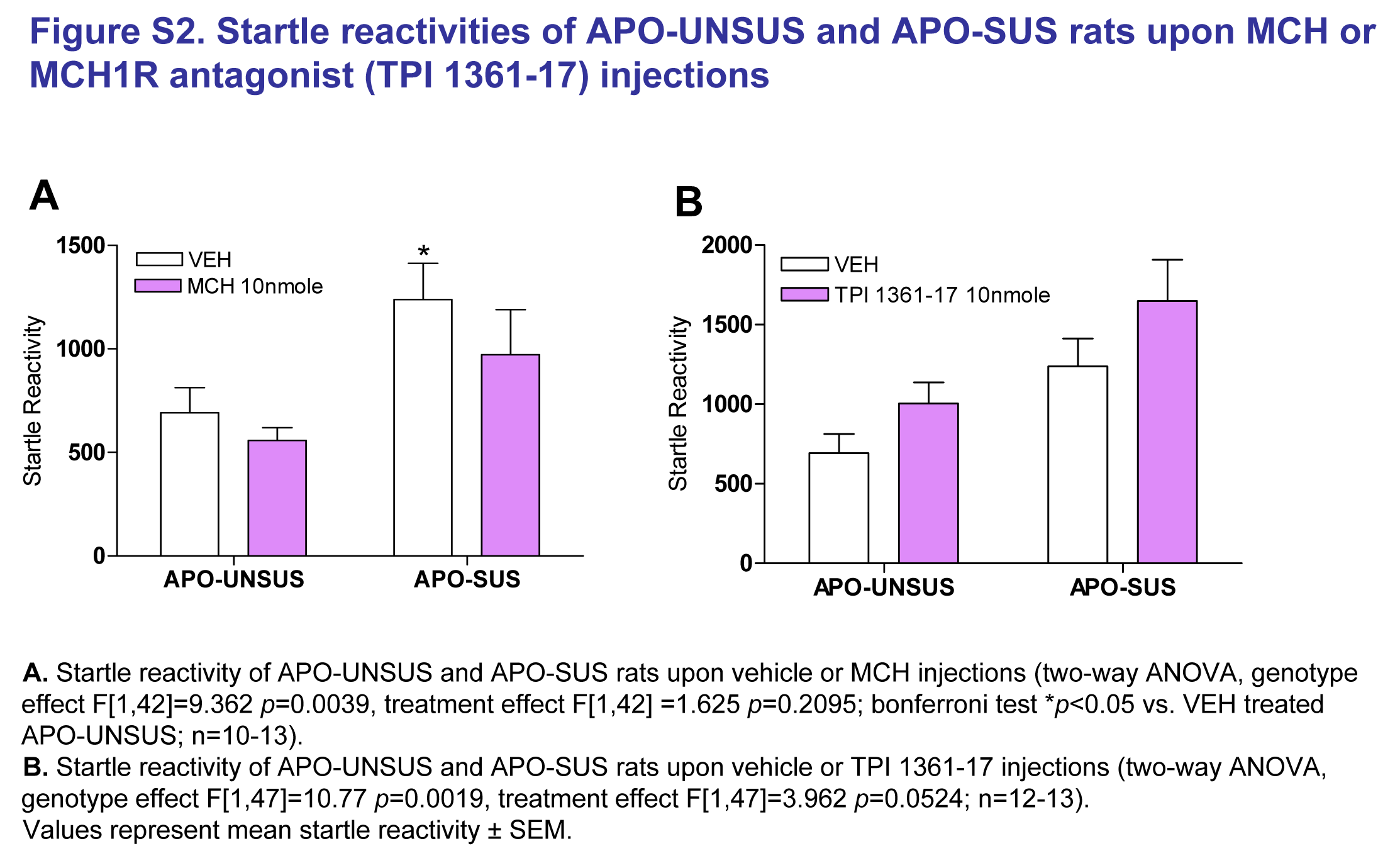

Supplement: Figure S2 — Startle reactivities of APO-UNSUS and APO-SUS rats upon MCH or MCH1R antagonist (TPI 1361-17) injections. A. Startle reactivity of APO-UNSUS and APO-SUS rats upon vehicle or MCH injections (two-way ANOVA, genotype effect F[1,42] = 9.362 p = 0.0039, treatment effect F[1,42] = 1.625 p = 0.2095; bonferroni test *p<0.05 vs. VEH treated APO-UNSUS; n = 10–13). B. Startle reactivity of APO-UNSUS and APO-SUS rats upon vehicle or TPI 1361-17 injections (two-way ANOVA, genotype effect F[1,47] = 10.77 p = 0.0019, treatment effect F[1,47] = 3.962 p = 0.0524; n = 12–13). Values represent mean startle reactivity ± SEM. (TIF) [file pone.0019286.s002.tif]

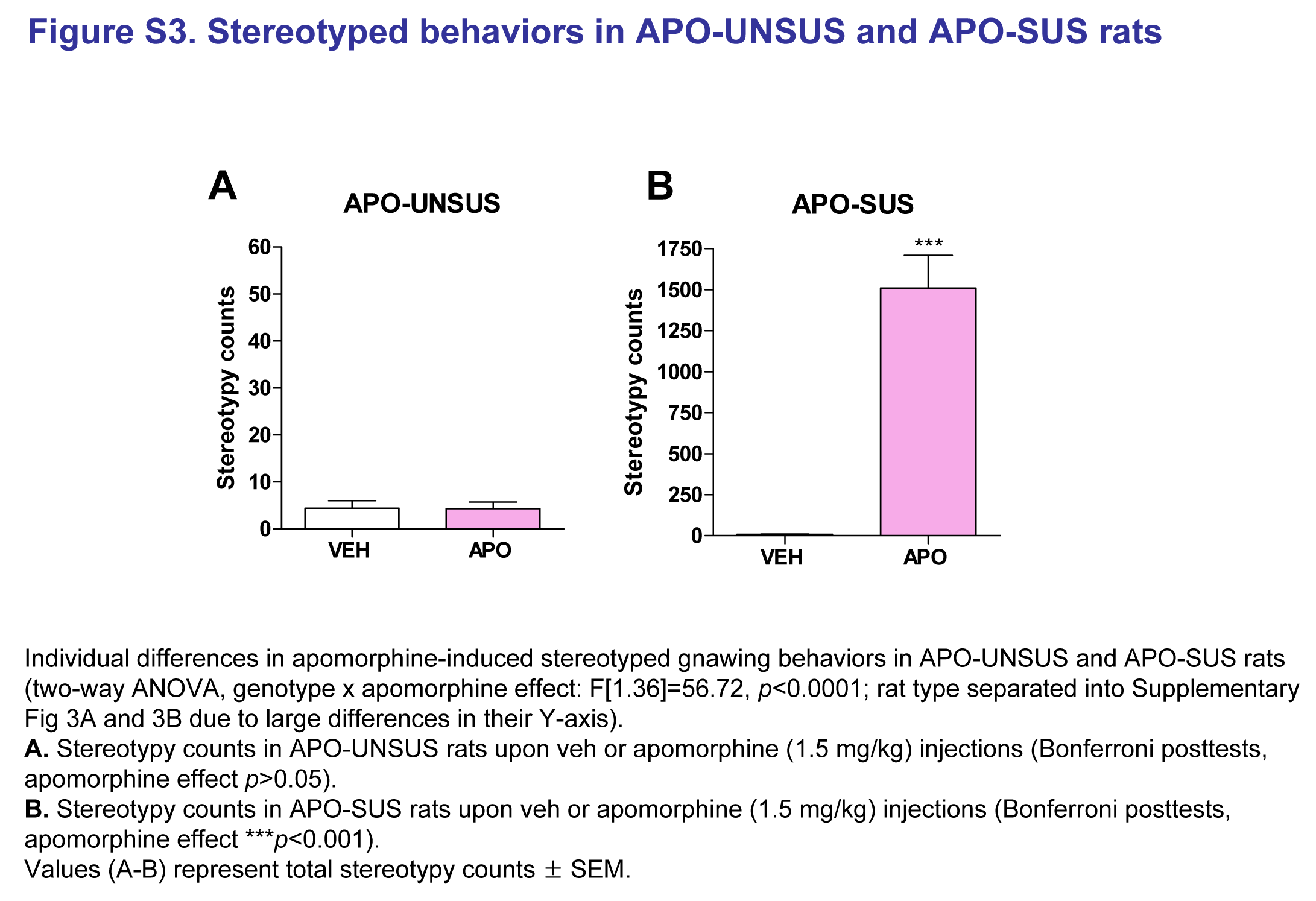

Supplement: Figure S3 — Stereotyped behaviors in APO-UNSUS and APO-SUS rats. Individual differences in apomorphine-induced stereotyped gnawing behaviors in APO-UNSUS and APO-SUS rats (two-way ANOVA, genotype×apomorphine effect: F[1.36] = 56.72, p<0.0001; rat type separated into Figure S3A and S3B due to large differences in their Y-axis). A. Stereotypy counts in APO-UNSUS rats upon veh or apomorphine (1.5 mg/kg) injections (Bonferroni posttests, apomorphine effect p>0.05). B. Stereotypy counts in APO-SUS rats upon veh or apomorphine (1.5 mg/kg) injections (Bonferroni posttests, apomorphine effect ***p<0.001). Values (A–B) represent total stereotypy counts ± SEM. (TIF) [file pone.0019286.s003.tif]

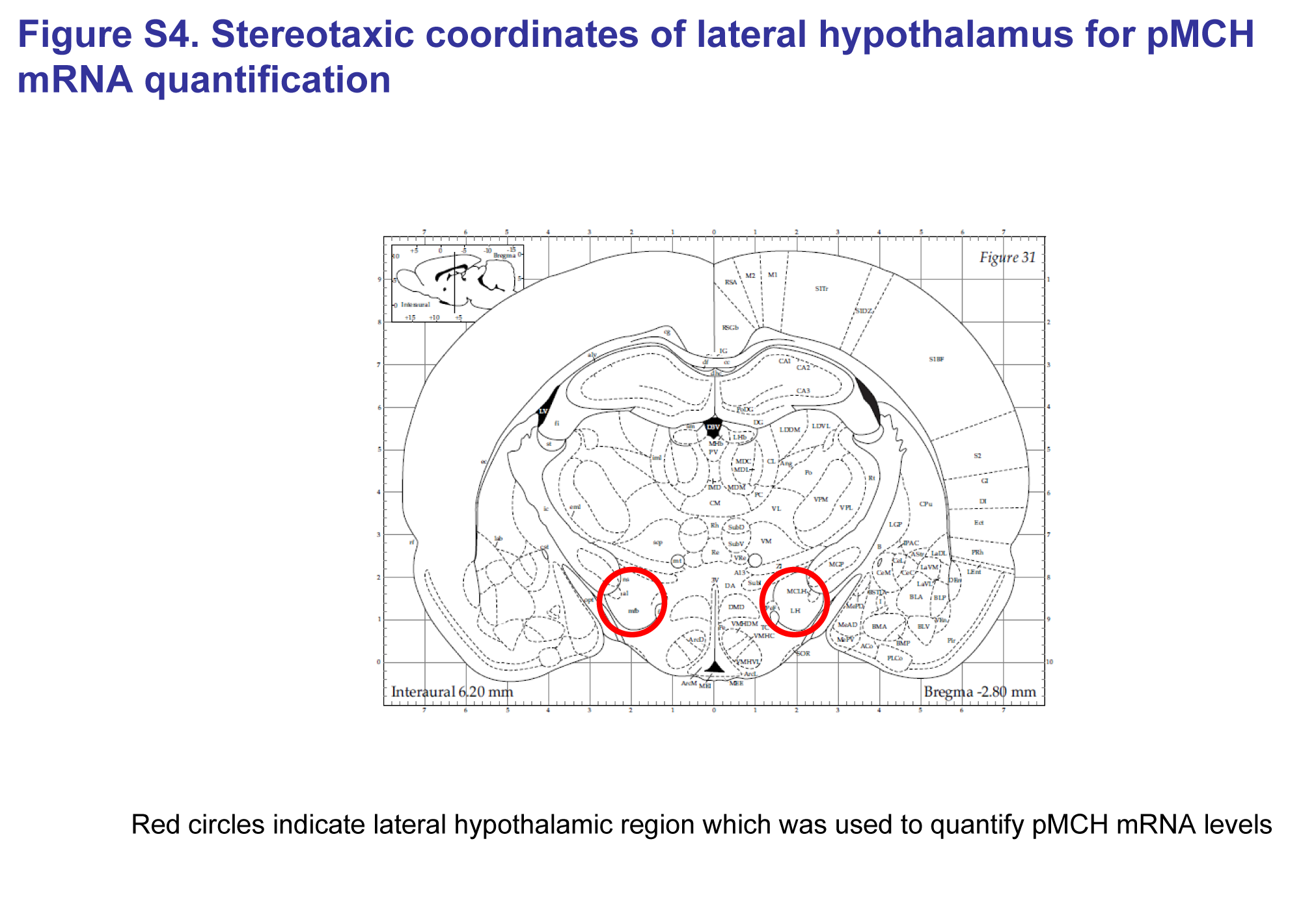

Supplement: Figure S4 — Stereotaxic coordinates of lateral hypothalamus for pMCH mRNA quantification. Red circles indicate lateral hypothalamic region which was used to quantify pMCH mRNA levels. (TIF) [file pone.0019286.s004.tif]

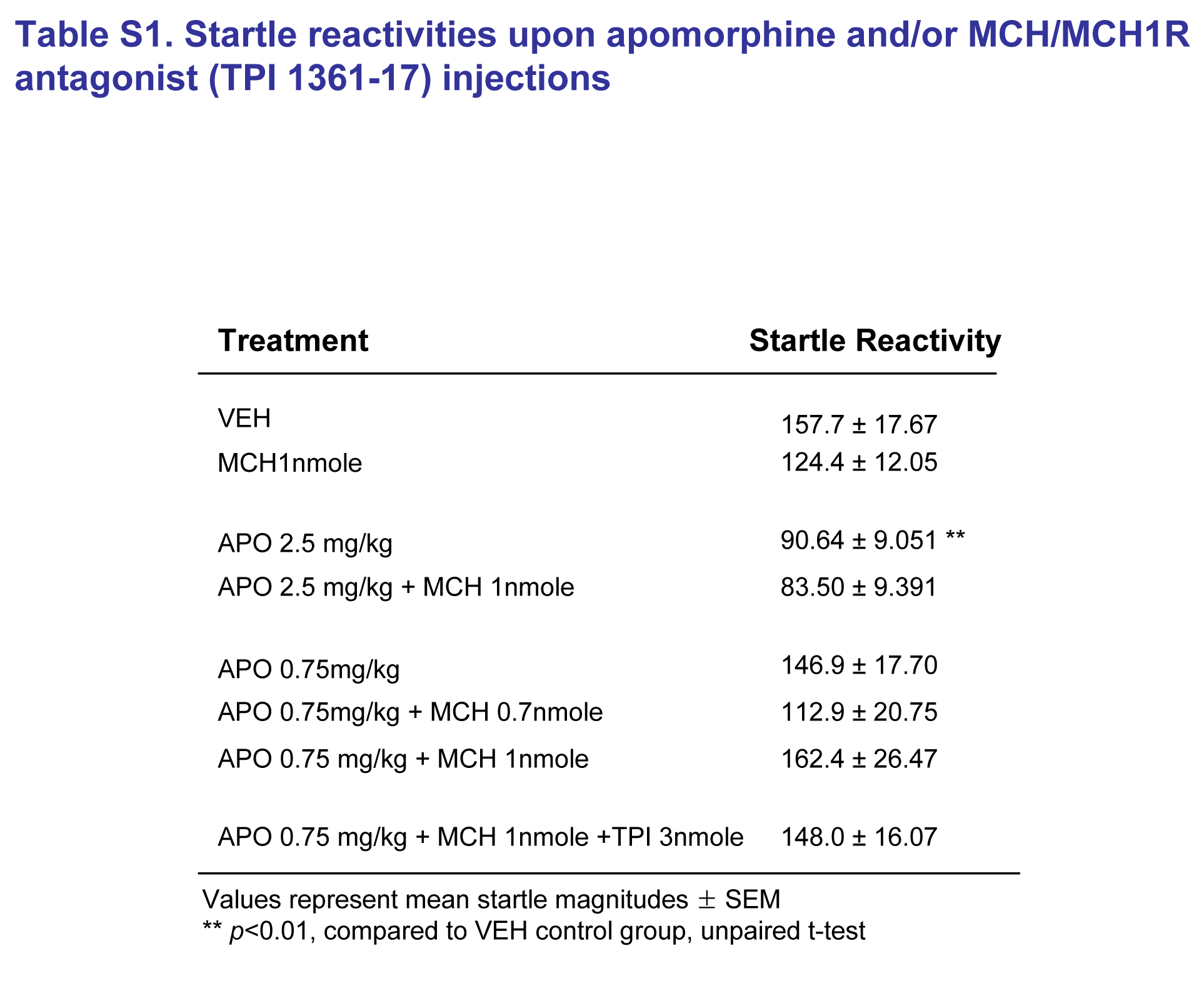

Supplement: Table S1 — Startle reactivities upon apomorphine and/or MCH/MCH1R antagonist (TPI 1361-17) injections. (TIF) [file pone.0019286.s005.tif]
